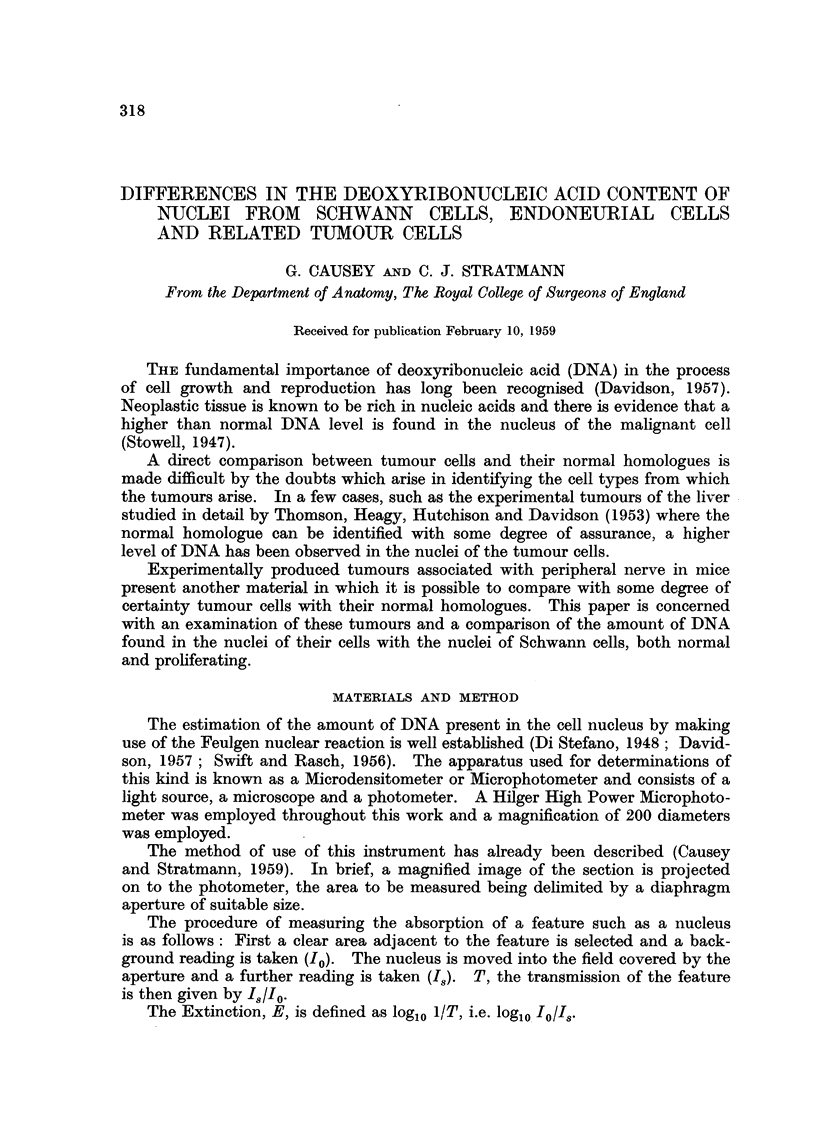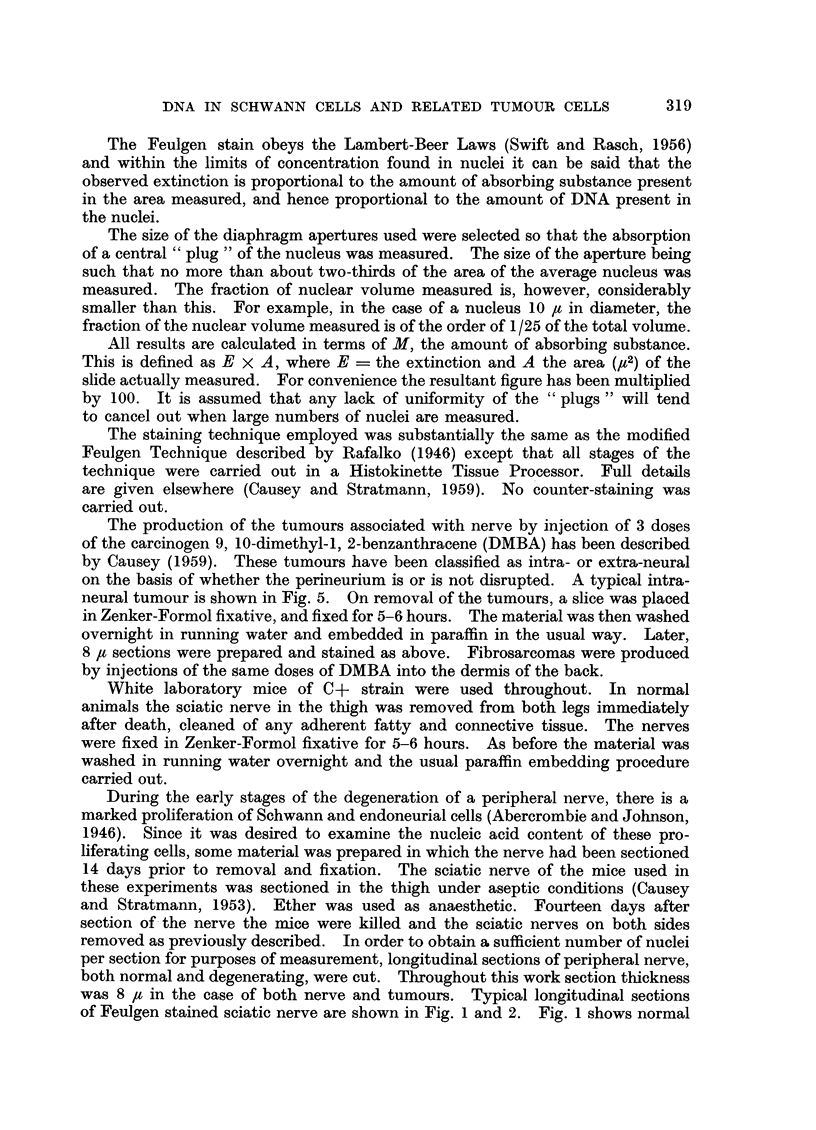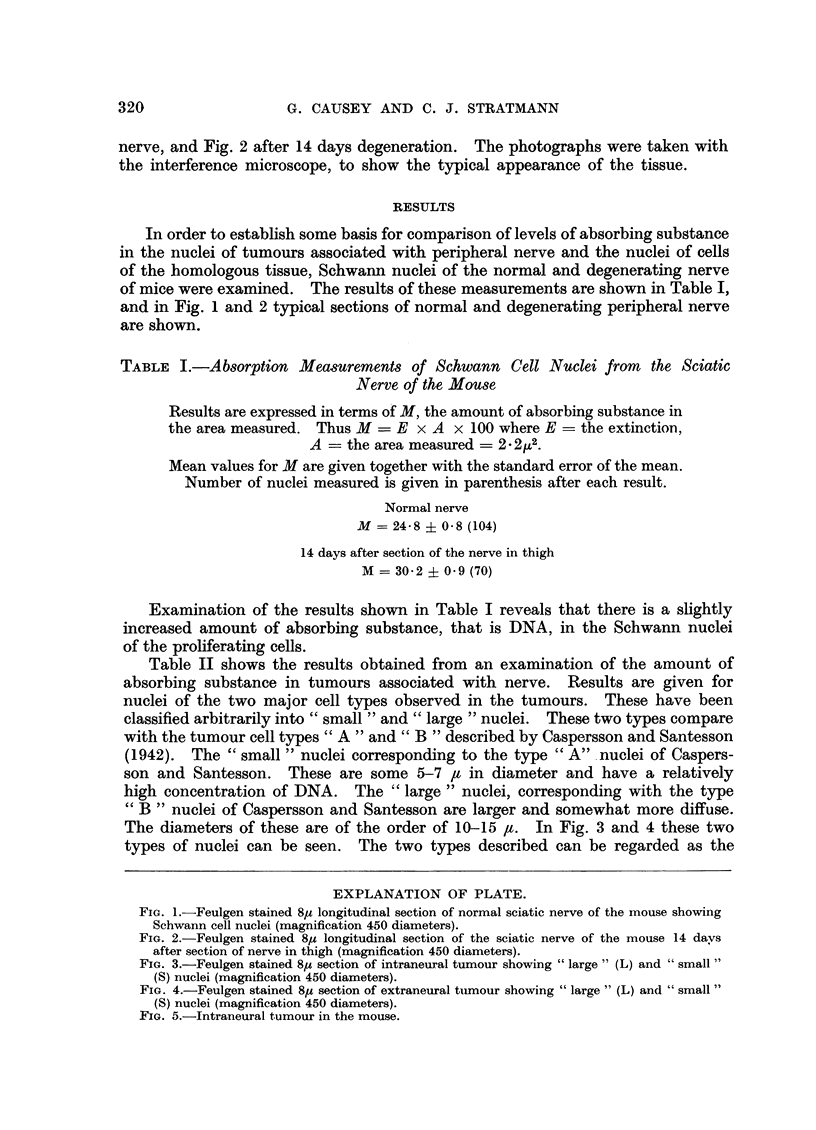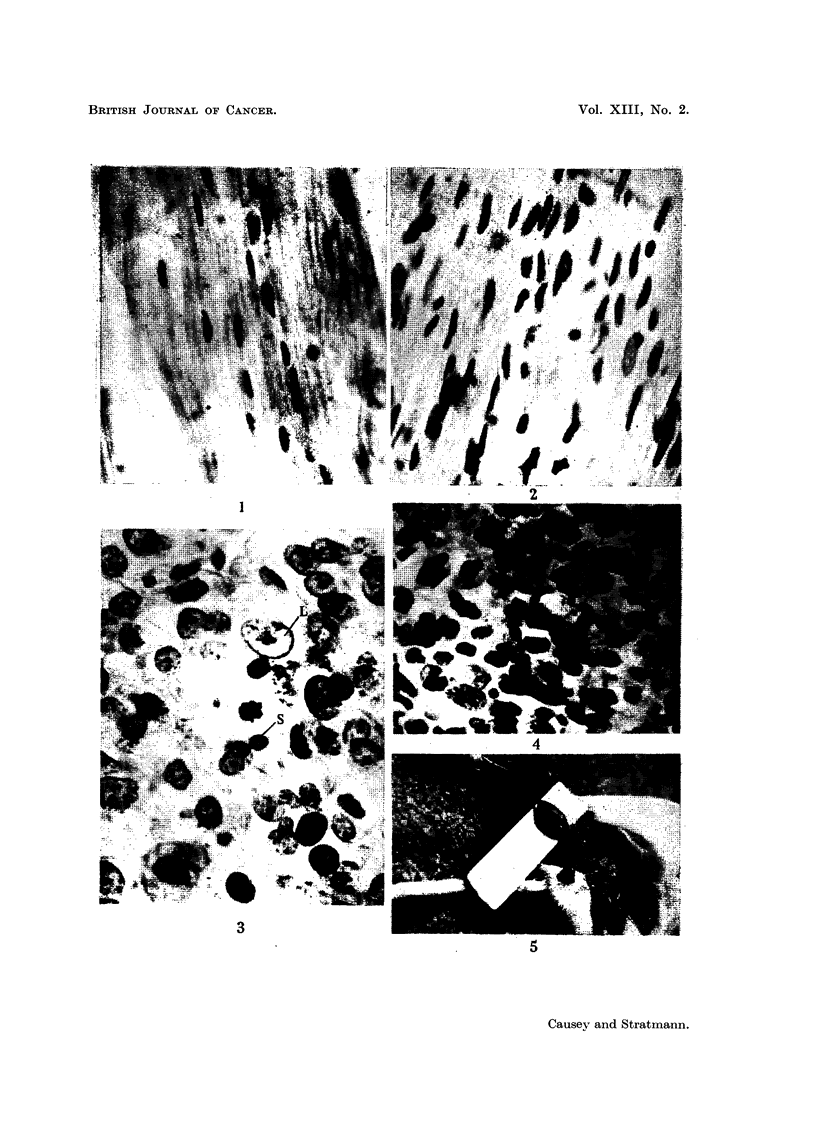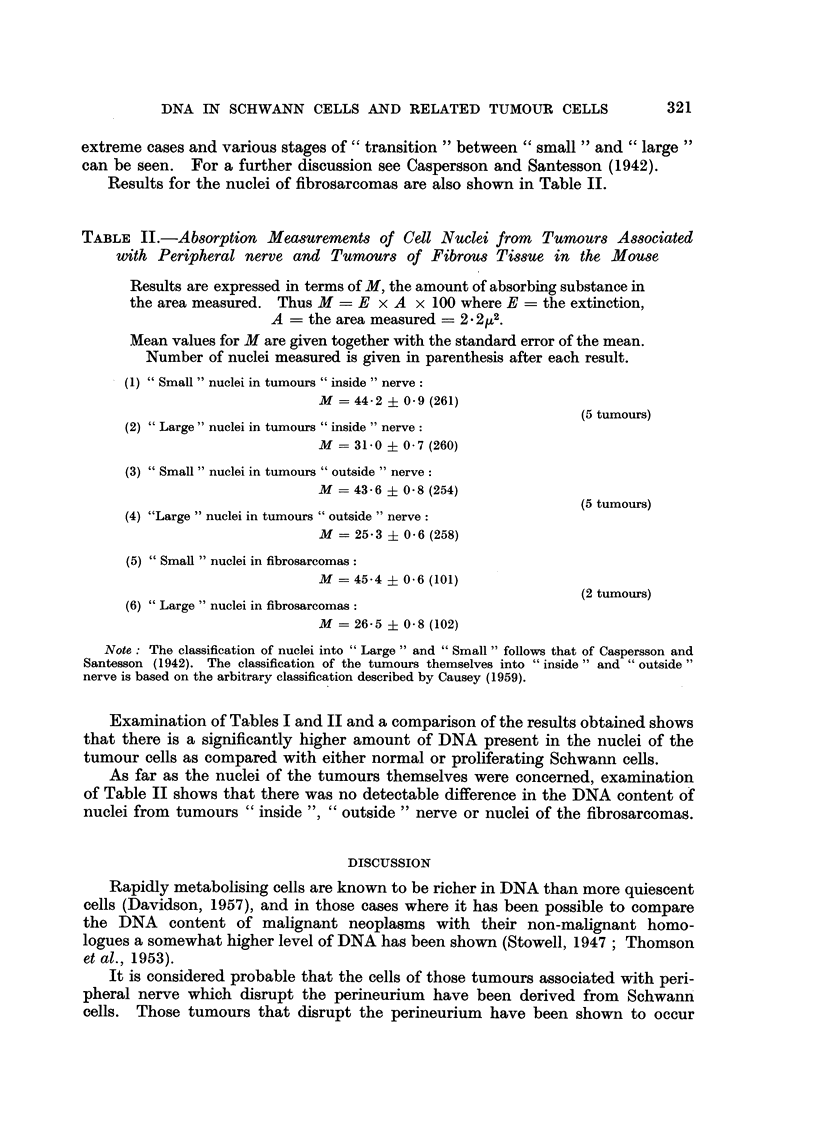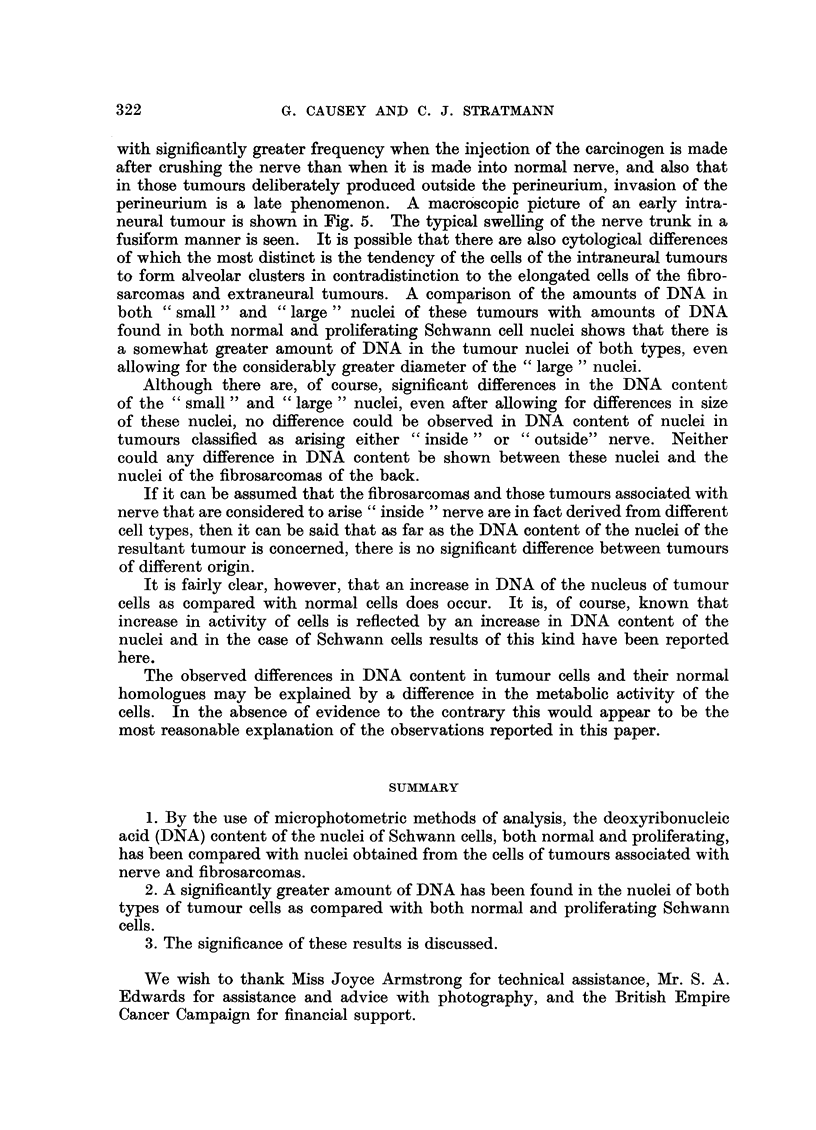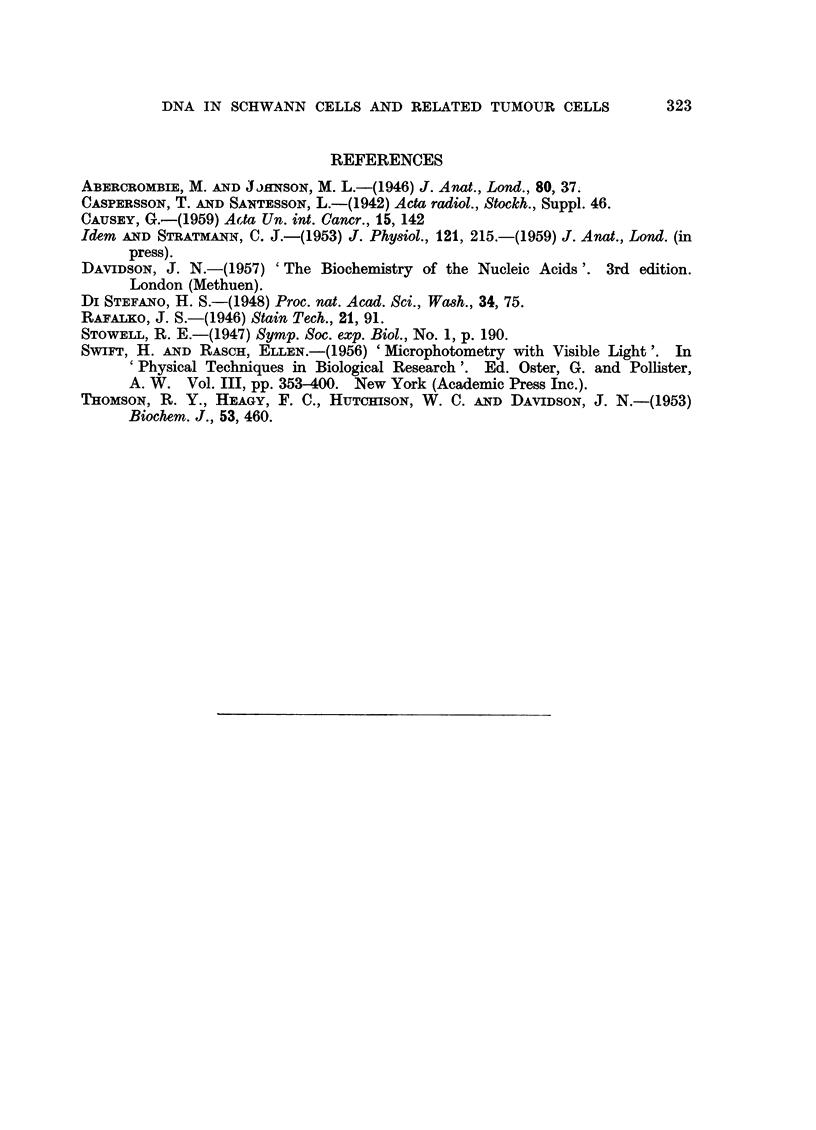# Differences in the Deoxyribonucleic Acid Content of Nuclei from Schwann Cells, Endoneurial Cells and Related Tumour Cells

**DOI:** 10.1038/bjc.1959.39

**Published:** 1959-06

**Authors:** G. Causey, C. J. Stratmann

## Abstract

**Images:**


### DIFFERENCES IN THE DEOXYRIBONUCLEIC ACID CONTENT OF NUCLEI FROM SCHWANN CELLS, ENDONEURIAL CELLS AND RELATED TUMOUR CELLS

G. CAUSEY AND C. J. STRATMANN

*From the Department of Anatomy, The Royal College of Surgeons of England*

Received for publication February 10, 1959

THE fundamental importance of deoxyribonucleic acid (DNA) in the process of cell growth and reproduction has long been recognised (Davidson, 1957). Neoplastic tissue is known to be rich in nucleic acids and there is evidence that a higher than normal DNA level is found in the nucleus of the malignant cell (Stowell, 1947).

A direct comparison between tumour cells and their normal homologues is made difficult by the doubts which arise in identifying the cell types from which the tumours arise. In a few cases, such as the experimental tumours of the liver studied in detail by Thomson, Heagy, Hutchison and Davidson (1953) where the normal homologue can be identified with some degree of assurance, a higher level of DNA has been observed in the nuclei of the tumour cells.

Experimentally produced tumours associated with peripheral nerve in mice present another material in which it is possible to compare with some degree of certainty tumour cells with their normal homologues. This paper is concerned with an examination of these tumours and a comparison of the amount of DNA found in the nuclei of their cells with the nuclei of Schwann cells, both normal and proliferating.

### MATERIALS AND METHOD

The estimation of the amount of DNA present in the cell nucleus by making use of the Feulgen nuclear reaction is well established (Di Stefano, 1948 ; Davidson, 1957 ; Swift and Rasch, 1956). The apparatus used for determinations of this kind is known as a Microdensitometer or Microphotometer and consists of a light source, a microscope and a photometer. A Hilger High Power Microphotometer was employed throughout this work and a magnification of 200 diameters was employed.

The method of use of this instrument has already been described (Causey and Stratmann, 1959). In brief, a magnified image of the section is projected on to the photometer, the area to be measured being delimited by a diaphragm aperture of suitable size.

The procedure of measuring the absorption of a feature such as a nucleus is as follows : First a clear area adjacent to the feature is selected and a background reading is taken ($I_0$). The nucleus is moved into the field covered by the aperture and a further reading is taken ($I_s$). $T$, the transmission of the feature is then given by $I_s/I_0$.

The Extinction, $E$, is defined as $\log_{10} 1/T$, i.e. $\log_{10} I_0/I_s$.

The Feulgen stain obeys the Lambert-Beer Laws (Swift and Rasch, 1956) and within the limits of concentration found in nuclei it can be said that the observed extinction is proportional to the amount of absorbing substance present in the area measured, and hence proportional to the amount of DNA present in the nuclei.

The size of the diaphragm apertures used were selected so that the absorption of a central " plug " of the nucleus was measured. The size of the aperture being such that no more than about two-thirds of the area of the average nucleus was measured. The fraction of nuclear volume measured is, however, considerably smaller than this. For example, in the case of a nucleus 10 $\mu$ in diameter, the fraction of the nuclear volume measured is of the order of 1/25 of the total volume.

All results are calculated in terms of $M$, the amount of absorbing substance. This is defined as $E \times A$, where $E =$ the extinction and $A$ the area ($\mu^2$) of the slide actually measured. For convenience the resultant figure has been multiplied by 100. It is assumed that any lack of uniformity of the " plugs " will tend to cancel out when large numbers of nuclei are measured.

The staining technique employed was substantially the same as the modified Feulgen Technique described by Rafalko (1946) except that all stages of the technique were carried out in a Histokinette Tissue Processor. Full details are given elsewhere (Causey and Stratmann, 1959). No counter-staining was carried out.

The production of the tumours associated with nerve by injection of 3 doses of the carcinogen 9, 10-dimethyl-1, 2-benzanthracene (DMBA) has been described by Causey (1959). These tumours have been classified as intra- or extra-neural on the basis of whether the perineurium is or is not disrupted. A typical intraneural tumour is shown in Fig. 5. On removal of the tumours, a slice was placed in Zenker-Formol fixative, and fixed for 5–6 hours. The material was then washed overnight in running water and embedded in paraffin in the usual way. Later, 8 $\mu$ sections were prepared and stained as above. Fibrosarcomas were produced by injections of the same doses of DMBA into the dermis of the back.

White laboratory mice of C+ strain were used throughout. In normal animals the sciatic nerve in the thigh was removed from both legs immediately after death, cleaned of any adherent fatty and connective tissue. The nerves were fixed in Zenker-Formol fixative for 5–6 hours. As before the material was washed in running water overnight and the usual paraffin embedding procedure carried out.

During the early stages of the degeneration of a peripheral nerve, there is a marked proliferation of Schwann and endoneurial cells (Abercrombie and Johnson, 1946). Since it was desired to examine the nucleic acid content of these proliferating cells, some material was prepared in which the nerve had been sectioned 14 days prior to removal and fixation. The sciatic nerve of the mice used in these experiments was sectioned in the thigh under aseptic conditions (Causey and Stratmann, 1953). Ether was used as anaesthetic. Fourteen days after section of the nerve the mice were killed and the sciatic nerves on both sides removed as previously described. In order to obtain a sufficient number of nuclei per section for purposes of measurement, longitudinal sections of peripheral nerve, both normal and degenerating, were cut. Throughout this work section thickness was 8 $\mu$ in the case of both nerve and tumours. Typical longitudinal sections of Feulgen stained sciatic nerve are shown in Fig. 1 and 2. Fig. 1 shows normal nerve, and Fig. 2 after 14 days degeneration. The photographs were taken with the interference microscope, to show the typical appearance of the tissue.

### RESULTS

In order to establish some basis for comparison of levels of absorbing substance in the nuclei of tumours associated with peripheral nerve and the nuclei of cells of the homologous tissue, Schwann nuclei of the normal and degenerating nerve of mice were examined. The results of these measurements are shown in Table I, and in Fig. 1 and 2 typical sections of normal and degenerating peripheral nerve are shown.

TABLE I.—*Absorption Measurements of Schwann Cell Nuclei from the Sciatic Nerve of the Mouse*

Results are expressed in terms of $M$, the amount of absorbing substance in the area measured. Thus $M = E \times A \times 100$ where $E =$ the extinction, $A =$ the area measured $= 2 \cdot 2\mu^2$.

Mean values for $M$ are given together with the standard error of the mean. Number of nuclei measured is given in parenthesis after each result.

Normal nerve

$M = 24 \cdot 8 \pm 0 \cdot 8$ (104)

14 days after section of the nerve in thigh
$M = 30 \cdot 2 \pm 0 \cdot 9$ (70)

Examination of the results shown in Table I reveals that there is a slightly increased amount of absorbing substance, that is DNA, in the Schwann nuclei of the proliferating cells.

Table II shows the results obtained from an examination of the amount of absorbing substance in tumours associated with nerve. Results are given for nuclei of the two major cell types observed in the tumours. These have been classified arbitrarily into " small " and " large " nuclei. These two types compare with the tumour cell types " A " and " B " described by Caspersson and Santesson (1942). The " small " nuclei corresponding to the type " A" nuclei of Caspersson and Santesson. These are some 5–7 $\mu$ in diameter and have a relatively high concentration of DNA. The " large " nuclei, corresponding with the type " B " nuclei of Caspersson and Santesson are larger and somewhat more diffuse. The diameters of these are of the order of 10–15 $\mu$. In Fig. 3 and 4 these two types of nuclei can be seen. The two types described can be regarded as the

---

EXPLANATION OF PLATE.

FIG. 1.—Feulgen stained $8\mu$ longitudinal section of normal sciatic nerve of the mouse showing Schwann cell nuclei (magnification 450 diameters).
FIG. 2.—Feulgen stained $8\mu$ longitudinal section of the sciatic nerve of the mouse 14 days after section of nerve in thigh (magnification 450 diameters).
FIG. 3.—Feulgen stained $8\mu$ section of intraneural tumour showing " large " (L) and " small " (S) nuclei (magnification 450 diameters).
FIG. 4.—Feulgen stained $8\mu$ section of extraneural tumour showing " large " (L) and " small " (S) nuclei (magnification 450 diameters).
FIG. 5.—Intraneural tumour in the mouse.

1

2

3

4

5

extreme cases and various stages of " transition " between " small " and " large " can be seen. For a further discussion see Caspersson and Santesson (1942).

Results for the nuclei of fibrosarcomas are also shown in Table II.

TABLE II.—*Absorption Measurements of Cell Nuclei from Tumours Associated with Peripheral nerve and Tumours of Fibrous Tissue in the Mouse*

Results are expressed in terms of $M$, the amount of absorbing substance in the area measured. Thus $M = E \times A \times 100$ where $E$ = the extinction, $A$ = the area measured = $2 \cdot 2\mu^2$.

Mean values for $M$ are given together with the standard error of the mean. Number of nuclei measured is given in parenthesis after each result.

| | | |
|---|---|---|
| (1) " Small " nuclei in tumours " inside " nerve : | $M = 44 \cdot 2 \pm 0 \cdot 9$ (261) | (5 tumours) |
| (2) " Large " nuclei in tumours " inside " nerve : | $M = 31 \cdot 0 \pm 0 \cdot 7$ (260) | |
| (3) " Small " nuclei in tumours " outside " nerve : | $M = 43 \cdot 6 \pm 0 \cdot 8$ (254) | (5 tumours) |
| (4) "Large " nuclei in tumours " outside " nerve : | $M = 25 \cdot 3 \pm 0 \cdot 6$ (258) | |
| (5) " Small " nuclei in fibrosarcomas : | $M = 45 \cdot 4 \pm 0 \cdot 6$ (101) | (2 tumours) |
| (6) " Large " nuclei in fibrosarcomas : | $M = 26 \cdot 5 \pm 0 \cdot 8$ (102) | |

*Note :* The classification of nuclei into " Large " and " Small " follows that of Caspersson and Santesson (1942). The classification of the tumours themselves into " inside " and " outside " nerve is based on the arbitrary classification described by Causey (1959).

Examination of Tables I and II and a comparison of the results obtained shows that there is a significantly higher amount of DNA present in the nuclei of the tumour cells as compared with either normal or proliferating Schwann cells.

As far as the nuclei of the tumours themselves were concerned, examination of Table II shows that there was no detectable difference in the DNA content of nuclei from tumours " inside ", " outside " nerve or nuclei of the fibrosarcomas.

### DISCUSSION

Rapidly metabolising cells are known to be richer in DNA than more quiescent cells (Davidson, 1957), and in those cases where it has been possible to compare the DNA content of malignant neoplasms with their non-malignant homologues a somewhat higher level of DNA has been shown (Stowell, 1947 ; Thomson *et al.*, 1953).

It is considered probable that the cells of those tumours associated with peripheral nerve which disrupt the perineurium have been derived from Schwann cells. Those tumours that disrupt the perineurium have been shown to occur with significantly greater frequency when the injection of the carcinogen is made after crushing the nerve than when it is made into normal nerve, and also that in those tumours deliberately produced outside the perineurium, invasion of the perineurium is a late phenomenon. A macroscopic picture of an early intraneural tumour is shown in Fig. 5. The typical swelling of the nerve trunk in a fusiform manner is seen. It is possible that there are also cytological differences of which the most distinct is the tendency of the cells of the intraneural tumours to form alveolar clusters in contradistinction to the elongated cells of the fibrosarcomas and extraneural tumours. A comparison of the amounts of DNA in both " small " and " large " nuclei of these tumours with amounts of DNA found in both normal and proliferating Schwann cell nuclei shows that there is a somewhat greater amount of DNA in the tumour nuclei of both types, even allowing for the considerably greater diameter of the " large " nuclei.

Although there are, of course, significant differences in the DNA content of the " small " and " large " nuclei, even after allowing for differences in size of these nuclei, no difference could be observed in DNA content of nuclei in tumours classified as arising either " inside " or " outside" nerve. Neither could any difference in DNA content be shown between these nuclei and the nuclei of the fibrosarcomas of the back.

If it can be assumed that the fibrosarcomas and those tumours associated with nerve that are considered to arise " inside " nerve are in fact derived from different cell types, then it can be said that as far as the DNA content of the nuclei of the resultant tumour is concerned, there is no significant difference between tumours of different origin.

It is fairly clear, however, that an increase in DNA of the nucleus of tumour cells as compared with normal cells does occur. It is, of course, known that increase in activity of cells is reflected by an increase in DNA content of the nuclei and in the case of Schwann cells results of this kind have been reported here.

The observed differences in DNA content in tumour cells and their normal homologues may be explained by a difference in the metabolic activity of the cells. In the absence of evidence to the contrary this would appear to be the most reasonable explanation of the observations reported in this paper.

### SUMMARY

1. By the use of microphotometric methods of analysis, the deoxyribonucleic acid (DNA) content of the nuclei of Schwann cells, both normal and proliferating, has been compared with nuclei obtained from the cells of tumours associated with nerve and fibrosarcomas.

2. A significantly greater amount of DNA has been found in the nuclei of both types of tumour cells as compared with both normal and proliferating Schwann cells.

3. The significance of these results is discussed.

We wish to thank Miss Joyce Armstrong for technical assistance, Mr. S. A. Edwards for assistance and advice with photography, and the British Empire Cancer Campaign for financial support.

### REFERENCES

ABERCROMBIE, M. AND JOHNSON, M. L.—(1946) *J. Anat., Lond.*, **80**, 37.
CASPERSSON, T. AND SANTESSON, L.—(1942) *Acta radiol., Stockh.*, Suppl. 46.
CAUSEY, G.—(1959) *Acta Un. int. Cancr.*, **15**, 142
*Idem* AND STRATMANN, C. J.—(1953) *J. Physiol.*, **121**, 215.—(1959) *J. Anat., Lond.* (in press).
DAVIDSON, J. N.—(1957) 'The Biochemistry of the Nucleic Acids'. 3rd edition. London (Methuen).
DI STEFANO, H. S.—(1948) *Proc. nat. Acad. Sci., Wash.*, **34**, 75.
RAFALKO, J. S.—(1946) *Stain Tech.*, **21**, 91.
STOWELL, R. E.—(1947) *Symp. Soc. exp. Biol.*, No. 1, p. 190.
SWIFT, H. AND RASCH, ELLEN.—(1956) 'Microphotometry with Visible Light'. In 'Physical Techniques in Biological Research'. Ed. Oster, G. and Pollister, A. W. Vol. III, pp. 353–400. New York (Academic Press Inc.).
THOMSON, R. Y., HEAGY, F. C., HUTCHISON, W. C. AND DAVIDSON, J. N.—(1953) *Biochem. J.*, **53**, 460.